# Interplay of silymarin and clove fruit extract effectively enhances cadmium stress tolerance in wheat (*Triticum aestivum*)

**DOI:** 10.3389/fpls.2023.1144319

**Published:** 2023-04-14

**Authors:** Ahmed H. El-Sappah, Mohamed A. S. Metwally, Mostafa M. Rady, Hayssam M. Ali, Linghui Wang, Pulak Maitra, Muhammad Ihtisham, Kuan Yan, Xin Zhao, Jia Li, El-Sayed M. Desoky

**Affiliations:** ^1^ School of Agriculture, Forestry, and Food Engineering, Yibin University, Yibin, Sichuan, China; ^2^ Genetics Department, Faculty of Agriculture, Zagazig University, Zagazig, Egypt; ^3^ Soil Science Department, Faculty of Agriculture, Zagazig University, Zagazi, Egypt; ^4^ Botany Department, Faculty of Agriculture, Fayoum University, Fayoum, Egypt; ^5^ Department of Botany and Microbiology, College of Science, King Saud University, Riyadh, Saudi Arabia; ^6^ Institute of Dendrology, Polish Academy of Sciences, Kórnik, Poland; ^7^ Botany Department, Faculty of Agriculture, Zagazig University, Zagazig, Egypt

**Keywords:** antioxidant system, heavy metals, osmoprotectants, plant growth regulators, plant stress

## Abstract

**Introduction:**

Osmoprotectant supplementation can be used as a useful approach to enhance plant stress tolerance. However, the effect of silymarin and clove fruit extract (CFE) on wheat plants grown under cadmium (Cd) stress has not been studied.

**Methods:**

Wheat seeds were planted in plastic pots filled with ions-free sand. A ½-strength Hoagland’s nutrient solution was used for irrigation. Pots were treated with eight treatments thirteen days after sowing: 1) Control, 2) 0.5 mM silymarin foliar application [silymarin], 3) 2% CFE foliar application [CFE], 4) CFE enriched with silymarin (0.24 g silymarin L-1 of CFE) [CFE-silymarin], 5) Watering wheat seedlings with a nutritious solution of 2 mM Cd [Cd]. 6) Cadmium + silymarin, 7) Cadmium + CFE, and 8) Cadmium + CFE-silymarin. The experimental design was a completely randomized design with nine replicates.

**Results and discussion:**

The Cd stress decreased grain yield, shoot dry weight, leaf area, carotenoids, chlorophylls, stomatal conductance, net photosynthetic rate, transpiration rate, membrane stability index, nitrogen, phosphorus, and potassium content by 66.9, 60.6, 56.7, 23.8, 33.5, 48.1, 41.2, 48.7, 42.5, 24.1, 39.9, and 24.1%, respectively. On the other hand, Cd has an Application of CFE, silymarin, or CEF-silymarin for wheat plants grown under Cd stress, significantly improved all investigated biochemical, morphological, and physiological variables and enhanced the antioxidant enzyme activities. Applying CFE and/or silymarin enhanced plant tolerance to Cd stress more efficiently. Our findings suggest using CFE-silymarin as a meaningful biostimulator for wheat plants to increase wheat plants’ tolerance to Cd stress *via* enhancing various metabolic and physiological processes.

## 1 Introduction

Wheat (*Triticum aestivum*) is one of the most important grain crops for food safety worldwide (Li et al., 2022). It is considered the most stable staple food resource, supplying the world population with a considerable proportion of the required calories (Desoky et al., 2021a). Annually, the world produces about 600 million tons grown on only 200 million hectares (Sowell and Swearingen, 2022). Since wheat is counted as the primary food crop in most regions of the world, advanced and effective methods must be followed to mitigate the harmful impacts of stressors on wheat production (Mansour et al., 2020). With urbanization and industrialization in the modern era, heavy metal (HM) pollution has been a prime obstacle to sustainable agricultural development (El-Sappah and Rather, 2022). Soil or water contamination with HM has expanded due to long-term waste recycling, industrial activities, sewage-based irrigation, and agrochemicals threatening human health and food security (Desoky et al., 2020a). Plants cultivated in HM-affected soil lead to the accumulation of such toxic metals in plant tissues, which causes morphological deformation and physio-biochemical disorders, resulting in severe damage during plant growth and development (Tiwari and Lata, 2018; El-Sappah et al., 2021b; Rady et al., 2023).

Cadmium (Cd) is not an essential nutrient for plants which was identified as a highly phytotoxic HM (Haider et al., 2021; Abbas et al., 2022.), as well as being a highly water-soluble element makes plants absorb it easily (Desoky et al., 2019a; 2019b). It replaces calcium (Ca) due to its similar chemical behavior, radius, and ionic charges. The toxicity of Cd aggravates the hyper-accumulation of the different reactive oxygen species (ROS;e.g., O^2−^, H_2_O_2_, and OH^−^), exacerbating cell redox homeostasis disruption and abnormalities to the plant organelles structures and cellular membranes (Yadav et al., 2012; Haider et al., 2022). Moreover, Cd could severely change many enzyme activities, including those involved in photosynthesis, carbon dioxide fixation, nitrogen (N), phosphorus (P) and carbohydrate metabolism, and rubisco (Tiwari and Lata, 2018), as well as interfere with gene expression, and signalling processes (Srivastava et al., 2017). Therefore, long-term exposure to Cd could adversely affect plants by decreasing the photosynthetic pigments contents and photosynthetic efficiency and inhibiting plant growth (Haider et al., 2021). Cd exposure reduces plant physiological activity by decreasing tissue water content, transpiration, and stomatal conductance (Gill and Tuteja, 2010). As a stress-counteract mechanism, the plant developed antioxidative machinery, including enzymatic and nonenzymatic antioxidants to scavenge oxidative stress biomarkers (Alzahrani and Rady, 2019; El-Sappah et al., 2021a; Kumari et al., 2021). Nevertheless, throughout most cases, internal defense systems were insufficient to protect plants from stress (Abd El-mageed et al., 2022). Therefore, several approaches are required for promoting Cd tolerance in crops, such as plant growth biostimulants. Exogenous support biostimulants/elicitors, such as natural plant extracts, may raise the plant’s tolerance to Cd stress.

Silymarin is the primary bioactive substance extracted from all parts of the *S. marianum*; Milk thistle. plant (Ali et al., 2022). Silymarin comprises flavonolignans and a flavonoid mixture such as silibinin, silydianin, isosilychristin, isosilybin, silychristin, and taxifolin (Marmouzi et al., 2021). Accumulating such active substance (i.e., silymarin) in plant tissues highly interacts with environmental stresses, having antioxidant properties, directly detoxifying and preventing ROS formation *via* inhibiting ROS production (Surai, 2015). As a result, silymarin or silymarin-fortified biostimulants have been used recently to help plants withstand environmental stresses (Ali et al., 2022). Additionally, silymarin helps maintain mitochondrial integrity and cell redox homeostasis by activating nonenzymatic and enzymatic antioxidants (Ali et al., 2022). Under severe water stress, milk thistle (silymarin) accumulates more silymarin and related components, associated with increased enzymatic antioxidants activity and chalcone synthase gene expression, inducing drought stress tolerance (Elsayed et al., 2019). Thus, exogenous support biostimulants like silymarin can potentially increase plant tolerance to Cd stress, but this is still only speculative.

Plant biostimulants are foliar fertilizers that modify the plant’s metabolic processes, significantly improving plant growth and production (European Biostimulants Industry Council, 2012; Mashamaite et al., 2022; Azzam et al., 2022). Some growth-promoting materials like antioxidants, osmoprotectants, phytohormones, and nutrients are found in plant extracts such as the seed extract of moringa (*Moringa oleifera* L.) and the root extract of licorice (*Glycyrrhiza glabra*) (Desoky et al. 2019a; Desoky et al. 2019b). These substances significantly strengthen the antioxidant defense systems in the plant to improve its ability to face different environmental stresses. Rady and Mohamed (2015) and Desoky et al. (2020b) reported that plant extracts significantly improve plant growth and productivity. Lately, Desoky et al. (2021b) have considered clove (*Syzygium aromaticum*) fruit extract (CFE) as one of these extracts. However, till now, scientists have not conducted any studies on using CFE as a bioactive stimulant to enhance plants’ tolerance to Cd stress. Furthermore, the integrative effect of silymarin and CFE on wheat plants grown under Cd stress has not been thoroughly studied.

The primary goal of this study was to determine how CFE, when combined with silymarin, improved plant physio-biochemistry, nonenzymatic antioxidants, enzyme activities, and developmental characteristics in Cd-stressed wheat plants. Because they contain high levels of osmoprotectants and antioxidants such as phenolic compounds, glutathione, ascorbic acid, -tocopherol (-TOC), salicylic acid, soluble sugars, proline, amino acids, selenium, and vitamins, the chemical composition of these biostimulants has the potential to improve plant performance (i.e., E, B, and A). They also have a high concentration of phytohormones such as zeatin-type cytokinins, gibberellins, auxins, and others. Thus, we hypothesize that the co-addition of CFE and silymarin will improve the wheat plant’s tolerance to Cd stress and ultimately increase wheat production. This hypothesis could be achieved by identifying a set of morpho-physio-biochemical attributes to study the effect of CFE and silymarin on wheat plants grown under Cd stress conditions.

## 2 Materials and methods

### 2.1 Materials, growth conditions, experimental treatments, and CFE preparation

Healthy grains of wheat (*Triticum aestivum* L., cv. Misr 2) were purchased from the Wheat Research Section, Agronomy Research Institute, Agriculture Research Centre, Giza, Egypt. For one-minute, wheat grain surfaces were sterilized utilizing 0.1% HgCl_2_. After that, seeds were swilled in deionized-sterilized water, air dried then implanted in plastic pots with a depth of 35 cm and diameter of 40 cm. The pots were filled with 10 kg and used as a growth medium. This sand was free from any cations or anions. Fifteen grains/pot were sown at equal distances and depths. After two weeks from sowing, seedlings were thinned to eight seedlings/pot. Under an open green-house condition, the experiments were conducted with 62.0–65.1% humidity and a day/night temperature of 19 ± 3/10 ± 2°C. Plants were irrigated using 0.5 strength Hoagland’s nutrient solution (Hoagland and Arnon, 1938). After an interval of every two days, a nutrient solution free from any stress treatments was used to moisten the soil in all pots to the field capacity up to full emergence. The cadmium (Cd; Sigma-Aldrich, St. Louis, MO, USA) procedure was performed one month after sowing. The preliminary study used Cd sulfate (CdSO4) as a Cd source to make a solution concentrated with 2 mM of Cd (Alzahrani et al., 2018).

Silymarin (Sigma-Aldrich, St. Louis, MO, USA) and CFE, or silymarin-enriched CFE, was added as a foliar application and sprayed seven days after the first addition Cd solution. The addition rates of CFE, silymarin, and silymarin-enriched CFE were 2%, 0.5 mM, or 0.24 g Sm L^-1^ of CFE, respectively. Seven and fourteen days later, another two foliar sprays were done. This timeline is based on our previous study (Alharby et al., 2020). A few drops of Tween-20(1%) were used as a surfactant to improve the spray solution adherence performance. Continuous measurements were performed to keep the Cd concentration at 2 mM using the Optima 3300DV ICP-MS instrument (Perkin-Elmer, Inductively Coupled Plasma, Waltham, Mass Spectrometer, MA 02451, USA). Thirty days after the initial Cd addition, the trials were accomplished. A completely randomized design (CRD) with nine replicates was done to arrange the trial pots. The following eight treatments were performed:

1- Control: no stress and foliar spray.
2- Silymarin: Foliar spray with 0.5 mM silymarin.
3-CFE: Foliar spray with 2% CFE.
4-CFE-silymarin: Foliar spray with CFE enriched with silymarin (0.24 g silymarinL^-1^ of CFE).
5-Cd: Watering wheat seedlings with a nutritious solution of 2 mM Cd.
6- Cd+ silymarin: Watering wheat seedlings with a nutritious 2 mM Cd + foliar spray solution with 0.5 mM silymarin.
7-Cd+CFE: Watering wheat seedlings with a nutritious solution of 2 mMCd + foliar spraywith 2% CFE.
8- Cd+CFE-silymarin:Watering wheat seedlings with a nutritious solution of 2 mM Cd + foliar spraywith CFE enriched with silymarin (0.24 g silymarinL^-1^ of CFE).

### 2.2 Preparation and analysis of clove fruit extract

Clove fruit was extracted by weighing 10 g of clove fruits, drying, and soaking it in a liter of water at 50°C for 24 hours and then filtering and supplementing the final volume to a liter. The chemical analysis of the extract is represented in **Table 1**.

**Table 1:** Chemical constituents of clove fruit extract (CFE) (on a dry weight basis).

Component	Value
1. Total phenolic compounds (TPC; mg GAE/g CFE)	324
2. Total flavonoids (TF; mg QE/g CFE)	34.7
3. Phenolic compounds (mg/g CFE)	
3,4-Dihydroxybenzoic-acid	0.74
Ellagic-acid	0.62
Eugenol	105
Eugenyl-acetate	86.4
Gallic-acid	18.3
Naphthalene	0.21
Tannic acid	0.78
Vanillin	1.49
4. Antioxidants and osmoprotectants:	
Total free amino acid(g/kg)	70.2
Free proline (mg/kg)	19.0
Soluble sugars (mg/kg)	55.6
5. Mineral nutrients (g/kg)	
Mg	3.20
Ca	12.3
Fe	1.30
P	11.8
K	16.5
N	16.9
6. Vitamins (mg/kg)	
Vitamin A	25.6
Vitamin E	55.2
Vitamin D	32.4
Vitamin C	36.9

#### 2.2.1 Determination of total phenolic compounds, total flavonoids of CFE

TPC content in CFE was estimated by a UV spectrophotometer (Jenway-UV–VIS Spectrophotometer 6705) because of a colorimetric reduction of the reagent by phenolic compounds utilizing the formation of a blue complex, as described by Škerget et al. (2005). The oxidative reagent used was the Folin-Ciocalteu reagent (AOAC, 1990). TPC quantity has been shown in **Table 1** as mg GAE g^−1^ extract. TF content was determined according to Ordonez et al. (2006). **Table 1** present the TF contents of the extract as mg quercetin equivalent/g extract (mg QE g^−1^).

#### 2.2.2 Antioxidant activity of CFE

**Figure 1:**
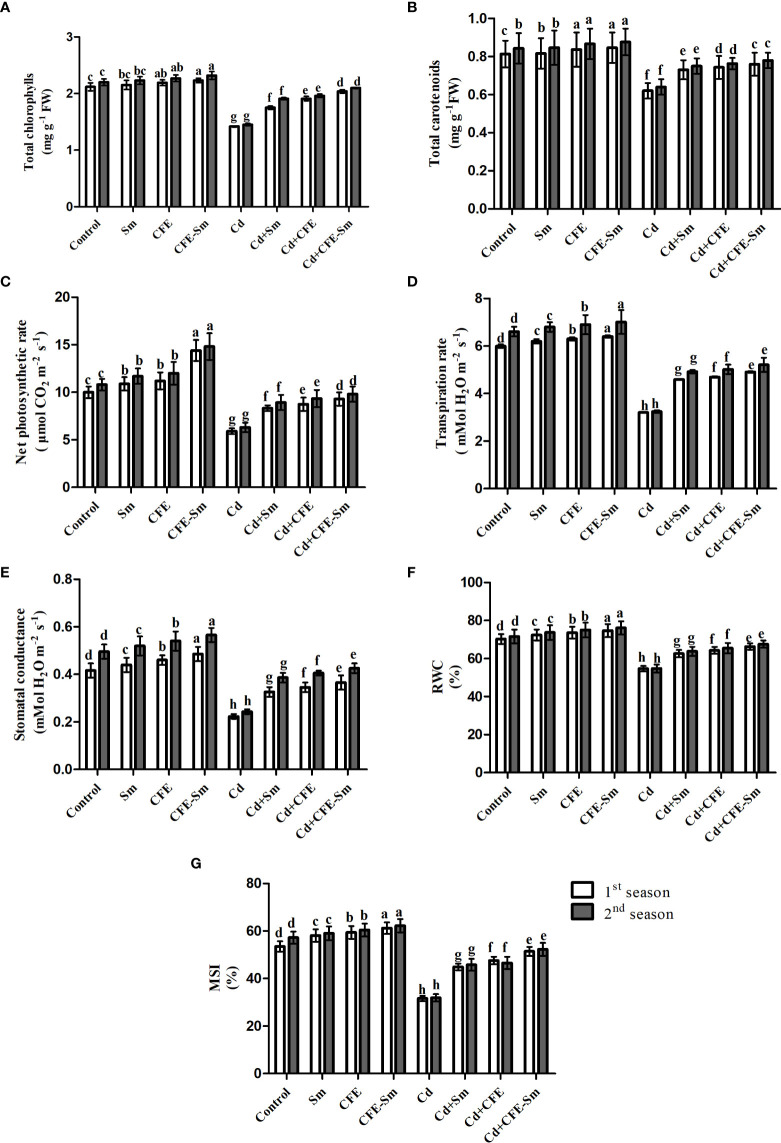
Physiological parameters of the wheat plant under Cd stress after foliar application of silymarin (Sm), clove fruit extract (CFE), or silymarin-enriched clove fruit extract (CFE-Sm). **(A)** total chlorophylls, **(B)** total carotenoids, **(C)** net photosynthetic rate, **(D)** Pn; transpiration rate (Tr); **(E)** Stomatal conductance (Gs), **(F)** relative water content (RWC), and **(G)** membrane stability index (MSI).

1, 1-Diphenyl-2 picrylhydrazyl (DPPH˙) *radical-antioxidant activity:* The electron donation ability of the extracts was measured by bleaching of the DPPH˙ purple-colored solution Hanato et al. (1988). β-Carotene/linoleic acid bleaching: The capacity of CFE and synthetic antioxidants (gallic acid and TBHQ) for preventing the β-carotene bleaching was evaluated according to Dastmalchi et al. (2007). Ferric reducing antioxidant power (FRAP): The FRAP was measured following a methodology described by Gülçin et al. (2010). Tert-butyl hydroquinone (TBHQ) and gallic acid were regarded as positive control of all three methods. Three replicates were analyzed for all samples (**Figure 1**).

#### 2.2.3 Determination of phenolic and flavonoid compounds by High-performance liquid chromatography

HPLC analysis was executed according to (Sati et al., 2020) with slight modifications using an Agilent Technologies 1100 series liquid chromatograph equipped with an autosampler (**Table 1**).

### 2.3 Wheat morphological traits

The plants were harvested after 35 days of Cd application. Samples were collected from each treatment to evaluate plant height (cm), leave area (cm^2^), and shoot’s dry weight (g plant^-1^). At the harvest phase, samples were collected from ten randomly selected plants to estimate 1000 grain weight (g), the number of grains per spike, and grain yield (g plant^-1^).

### 2.4 Determinations of chlorophyll content, PSII quantum yield, and CO_2_ fixation rate

According to Fadeel (1962), pure acetone was used to extract total carotenoids and total chlorophyll from fresh leaves. Absorbance readings were recorded at 480 nm, 645 nm, and 663 nm using a spectrophotometer (Beckman 640D, USA) to calculate the contents of pigmentsin mg g−1 leaf fresh weight by utilizing a portable photosynthesis system (LF6400XTR, LI-COR, USA) stomatal conductance (gs), leaf net photosynthetic rate (Pn) and rate of transpiration (Tr), were estimated. Measurements were done between 09:00−11:00 a.m.

### 2.5 Determinations of relative water content, membrane stability index, inorganic ions leaked, malondialdehyde, leaf soluble sugars, and proline

The method by Barrs and Weatherley (1962) was used to estimate the relative water content (RWC). The fresh weight (FW) of the leaves was measured, and the leaves were left drenched in water for 3 hours. Then, the turgid weight (TW) of the leaves was calculated. The samples were then dried in an oven at 80°C for 24 hours and weighed (DW). The RWC was determined using the following formula: RWC = [(FW − DW)/(TW − DW)] × 100.

The membrane stability index (MSI) was determined using 200 mg of a fresh leaf (two sets) in test tubes containing 10 cm^3^ of double-distilled water. One group of samples was heated at 40°C for 30 minutes. EC was recorded on a conductivity bridge (C1). The second group of samples was boiled at 100°C for ten minutes in a boiling water bath, and EC was measured (C2). As in the study by Premchandra et al. (1990), modified by Rady (2011), MSI was calculated using the following formula: MSI (%) = (1−[C1/C2]) × 100.

The method Sullivan and Ross (1979) described was used to measure the total ions seeped from leafy tissue. We measured the electrical conductivities (ECs) of the 20-leafy tissue disc solution three times, after 30 and 10 minutes of heating (45-55°C) and boiling (100°C), respectively. The following formula was utilized to compute EL:


EL(%)=[(EC2-EC1)/EC3]×100

Malondialdehyde (MDA) content (μmol g^−1^ FW) was measured in 0.1 g leaf homogenized in Na-phosphate buffer. The homogenate was centrifuged under cooling at 20,000 × g for 25 minutes. The supernatant was read at 532 nm and corrected for nonspecific turbidity at 600 nm, following the methods proposed by Heath and Packer (1968).

Total soluble sugars content was assessed: 0.2 g leaves were washed with 5 ml 70% ethanol and homogenized with 5 ml 96% ethanol. The extract was centrifuged at 3500 × g for 10 min. The supernatant was collected and stored at 4°C (Irigoyen et al., 1992). Freshly prepared anthrone (3 ml) was added to 0.1 ml supernatant. This mixture was incubated in a hot water bath for 10 min. The absorbance was recorded at 625 nm with a Bausch and Lomb- 2000 Spectronic Spectrophotometer

Bates et al. (1973) used the method to determine proline accumulation in bean leaves. Second fresh leaf material 0.1 g was ground with 10 ml of 3% (w/v) aqueous sulphosalicylic acid, and the homogenate was filtered through Whatman 2 filter paper. One milliliter of the filtrate was reacted with a one-milliliter acid ninhydrin reagent and one-milliliter glacial acetic acid in the test tube for one hour at 100°C. The reaction terminated in an ice bath. Two-milliliter Toluene was added to the mixture, and the upper toluene layer was measured absorbance at 520 nm using a UV spectrophotometer.

### 2.6 Evaluation of oxidative stress (hydrogen peroxide, and superoxide) and non-enzymatic antioxidant compounds

A 30 mM potassium phosphate buffer pH 7.4 + 8.4% H_2_PO_4_ + 2.5% TCA+ 0.3% FeCl_3_ + 0.8% bipyridyl was used as a constituent to receive the extract to determine the extract’s total content of Ascorbate AsA (µmol g^−1^ FW) (Kampfenkel and Van Montagu, 1995). The reaction was allowed to occur at 40oC and for only 30 minutes, and the readings were recorded at 525 nm absorbance. The method of Griffth (1980) was carried out to assess the content (µmol g^−1^ FW) of reduced and total glutathione (GSH). The content of GSH was estimated utilizing a unique reaction mixture consisting of leaf extract, sodium phosphate (7 mM) buffered at pH 6.8 with 5,5-dithiobis-(2-nitrobenzoic acid); DTNB (6 mM), and sodium phosphate (130 mM) buffered at pH 7.4. The reaction mixture was left for only 10 minutes at 30°C, and the readings were recorded at 412 nm absorbance. The α-tocopherol content (α-TOC; µmol g^–1^ of dry leaf weight) was assessed (Konings et al., 1996 and Ching and Mohamed, 2001). 0.02 gm of butylated hydroxytoluene (BHT) was weighted to be dissolved in900 mL of extraction solvent consisting of 100 L ofCH3–COO–CH2–CH3, n-hexane and n-hexane-ethyl acetate. The standard solutions (0.02 – 0.2 mg mL^–1^) were prepared using R-TOC with 0.05 gm 100 mL23er-=yh^–1^ n-hexane. Then, the content of α-TOC was evaluated utilizing HPLC systems with a mobile phase ratio of 94 methanol: 6 water(v/v) along with a flow rate of 1.5 mL/minute and setting the UV detector at 292 nm. H_2_O_2_ level (µmol g^–1^ leaf fresh weight) was estimated by homogenizing 0.25 gm of fresh leaves in 5mL of TCA (5%). At a temperature of 4°C and for only 15 minutes, the homogenate centrifugation at 12000 rpm was carried out. A standard solution made from H_2_O_2_ was used to calibrate the spectrophotometer to record the reading at a wavelength of 390 nm (Velikova et al., 2000).

The method explained by Kubis (2008) was followed to determine ${\text{O}}_{2}^{•-}$ , in which, into fragments with a size of 1 mm× 1 mm, a pea fully-expanded fresh leaf was divided. And then, at room temperature for only one hour, these fragments were submerged into 10 mM K-phosphate buffered at pH 7.8, 10 mM NaN_3,_ and 0.05% NBT. At 85°C and for only 15 minutes, 2 mL of the solution was heated and rapidly cooled. Colorimetrically, the optical concentration was evaluated at the nm level of A580 nm.

Hydrogen peroxide (H_2_O_2_) content (µmol g^−1^ FW) was determined in the acetone extract. After adding titanium reagent and ammonium, the extract was dissolved in 1 M H_2_SO_4;_ the absorbance was measured at 415 nm (Mukherjee and Choudhari, 1983). The dried powdered spinach leaves were weighed to estimate their contents of heavy metals, which were determined using atomic absorption spectrophotometry according to AOAC (1984).

### 2.7 Evaluating the activities of antioxidant enzymes

0.5 gm of fresh leaves was extracted following the method described by Mukherjee and Choudhari (1983). The extracts were frozen in liquid nitrogen (N) and ground in a 100 mM phosphate buffer at a pH of 7.0. At a temperature of 4°C and for only 10 minutes, the homogenate centrifugation at 15,000 rpm was performed (Benchmark Scientific LC-8 Place Rotor; 1500xg, USA). Then, at a temperature of 4°C, the supernatants were kept in order to estimate the activity of superoxide dismutase (SOD), peroxidase (POD), and catalase (CAT). Using the method defined by Giannopolitis and Ries (1977), which called the nitro blue tetrazolium (NBT), SOD (EC1.15.1.1) activity was estimated. To evaluate CAT (EC1.11.1.6) activity, the method detailed by Aebi (1984) was carried out, while methods explained by Maehly and Chance (1954) and Klapheck et al. (1990) were performed to evaluate POD (EC1.11.1.7) activity.

### 2.8 Determinations of mineral content

We digested 0.1 g of dried-powdered leaf for 12 h using a mixture of 10 mL concentrated H_2_SO_4_ and 2 mL perchloric acid (80%) (Wolf, 1982). Using a flame photometer, the total concentration of potassium (K) and calcium (Ca) was estimated (Lachica et al., 1973), while a microkjeldahl method was carried out to assess total N (Chapman and Pratt, 1982). Using the ascorbic acid method, total phosphorus (P) was measured following the method of Watanabe and Olsen (1965). The method detailed by Chapman and Pratt (1962) was used to measure the content by Cd utilizing the atomic absorption spectrophotometer (Perkin-Elmer, Model 3300).

### 2.9 Anatomical studies

Fifty-five days after the seedling, the median portion of the main stem leaflet was used to perform the comparative microscopy analysis. The solution of formalin-acetic acid-alcohol was utilized to fix the samples Paolillo and Zobel (2002). The preserved stem and leaflet were divided into 5 mm segments cut horizontally into thin cross sections. Johansen’s pigments were used to stain the cross sections Johansen, 1940. As a clearing solution, ethanol and xylene: methyl salicylate (1:2, v/v) (99% purity, Sigma-Aldrich, St. Louis, MO, USA) were used to clarify the samples for better images. Finally, brief observations were done under a microscope, and delicate images were obtained using the EVOS FL Cell Imaging System (Thermo Fisher Scientific).

### 2.10 Statistical analysis

Variance (ANOVA) was analyzed for randomized block design in three replicates. The mean differences between Silymarin and Clove Fruit Extract were compared using the least significant difference test at a p ≤ 0.01 significance level.

## 3 Results

### 3.1 The response of wheat plant growth, yield components, photosynthetic pigments, photosynthetic efficiency, and leaf water status to CFE and/or silymarin

In the absence of stress, plant growth parameters (i.e., plant height, shoot dry weight, and leaf area), yield components (i.e., No grain spike^-1^, 1000 grain weight, and grain yield plant^-1^), photosynthetic pigments (i.e., total chlorophylls and total carotenoids), photosynthetic efficiency (i.e., Pn, Tr, and Gs), and leaf water status (i.e., RWC and MSI) were enhanced significantly (p ≤ 0.05) under the treatment of 2% CFE or 0.5 mM silymarin. Compared to the standard control treatment, CFE enriched with silymarin was the most efficient treatment, enhancing plant height, shoot dry weight, leaf area, No grain spike, 1000 grain weight, grain yield, total chlorophylls, total carotenoids, Pn, Tr, Gs, RWC and MSI on average by 9.54, 13.74, 5.48, 8.25, 5.37, 18.66, 5.32, 3.98, 40.98, 6.35, 18.75, 6.27, and 11.56%, respectively (**Table 2**; **Figure 1**). Compared to the standard control, all of the above attributes markedly declined by 41.8, 60.6, 56.7, 55.9, 50.4, 66.3, 33.5, 23.8, 41.2, 48.7, 48.1, 22.7, and 42.5%, respectively the stressed control (0.5 mM Cd) treatment (**Table 2**; **Figure 1**). All of the above attributes were significantly (p ≤ 0.05) enhanced with the treatment of CFE and/or silymarin compared to the stressed control, whereas CFE-silymarin treatment was the most efficient treatment, as all of the above attributes enhanced by 60.4, 97.1, 86.7, 94.1, 87.2, 160.9, 44.2, 22.2, 56.2, 57.1, 69.7, 22.2, and 63.3%, respectively. CFE-silymarin treatment improved the morphological parameters, yield component, photosynthetic pigments, leaf photosynthetic efficiency, and leaf water status of Cd-stressed plants. Still, it did not reach the same level as the typical control plants (**Tables 2**, **S1**; **Figure 1**).

**Table 2:** Wheat plant growth parameters and yield components after foliar application of silymarin (Sm), clove fruit extract (CFE), and silymarin-enriched clove fruit extract (CFE-Sm) under Cd stress.

Treatment	Growth parameters			Yield components		
	Plant height (cm)	Shoot dry weight (g)	Leaf area (cm^2^)	No grain spike^–1^	1000 grain weight (g)	Grain yield plant^-1^(g)
1^st^ season						
Control	80.6 ± 2.3^c^	4.94 ± 0.44^d^	34.3 ± 1.6^c^	60.0 ± 3.2^c^	53.2 ± 3.5^c^	12.1 ± 1.1^d^
Sm	84.5 ± 4.1^b^	4.98 ± 0.43^c^	35.2 ± 2.1^b^	61.6 ± 3.6^b^	54.0 ± 3.6^c^	13.4 ± 1.3^c^
CFE	86.5 ± 4.9^a^	5.25 ± 0.45^b^	35.2 ± 2.2^b^	63.7 ± 4.2^a^	55.0 ± 3.1^b^	13.8 ± 1.4^b^
CFE-Sm	88.2 ± 4.6^a^	5.54 ± 0.42^a^	36.1 ± 1.6^a^	64.9 ± 4.1^a^	56.0 ± 3.2^a^	14.4 ± 1.2^a^
Cd	46.7 ± 1.3^g^	1.91 ± 0.12^h^	14.6 ± 1.1^g^	26.2 ± 2.2^g^	26.5 ± 3.3^g^	3.96 ± 0.11^h^
Cd+Sm	58.0 ± 2.5^f^	2.70 ± 0.13^g^	20.0 ± 1.2^f^	37.0 ± 3.1^f^	38.5 ± 2.5^f^	5.61 ± .16^g^
Cd+CFE	63.9 ± 3.6^e^	3.07 ± 0.18^f^	23.7 ± 1.3^e^	41.8 ± 3.1^e^	44.6 ± 3.8^e^	7.95 ± 0.21^f^
Cd+CFE-Sm	75.1 ± 3.9^d^	3.73 ± 0.16^e^	27.6 ± 1.5^d^	51.3 ± 3.9^d^	49.7 ± 3.4^d^	10.4 ± 0.98^e^
**ANOVAdf** **Foliar 7**	<0.001	<0.001	<0.001	<0.001	<0.001	<0.001
2^nd^ season						
Control	81.8 ± 4.7^c^	4.95 ± 0.32^d^	35.0 ± 1.8^c^	62.3 ± 4.8^b^	54.7 ± 3.4c	13.1 ± 1.3^d^
Sm	85.6 ± 4.3^b^	5.11 ± 0.33^c^	36.1 ± 2.5^b^	63.9 ± 4.6^b^	55.5 ± 4.1^c^	14.1 ± 1.5^c^
CFE	88.0 ± 3.9^a^	5.40 ± 0.25^b^	36.0 ± 2.1^b^	66.2 ± 4.8^a^	55.6 ± 4.2^b^	14.8 ± 1.4^b^
CFE-Sm	89.7 ± 4.2^a^	5.71 ± 0.31^a^	37.0 ± 2.2^a^	67.5 ± 4.7^a^	57.7 ± 3.9^a^	15.5 ± 1.6^a^
Cd	47.7 ± 1.8^g^	1.98 ± 0.12^h^	15.4 ± 1.6^g^	27.7 ± 2.2^f^	27.4 ± 2.6^g^	4.36 ± 0.13^h^
Cd+Sm	59.1 ± 1.7^f^	3.16 ± 0.11^g^	21.5 ± 1.7^f^	38.6 ± 2.6^e^	40.6 ± 3.1^f^	6.20 ± 0.32^g^
Cd+CFE	65.1 ± 2.3^e^	3.50 ± 0.15^f^	24.5 ± 1.2^e^	43.7 ± 3.9^d^	46.9 ± 3.9^e^	9.35 ± 0.68^f^
Cd+CFE-Sm	76.4 ± 2.8^d^	3.94 ± 0.21^e^	28.4 ± 1.3^d^	53.3 ± 3.4^c^	51.2 ± 2.9^d^	11.3 ± 0.93^e^
**ANOVAdf** **Foliar**	<0.001	<0.001	<0.001	<0.001	<0.001	<0.001

Data are means (n = 9) ± SE. The same letters in each column indicate no significant differences according to the LSD test (p≤ 0.05).Control: There is no stress and no foliar applications, Sm: Foliar spray with 0.5 mM silymarin, CFE: Foliar spray with 2% clove fruit extract, CFE-Sm: Foliar spray with clove fruit extract enriched with silymarin (0.24 g Sm L^-1^ of CFE), Cd^+^: Watering the wheat seedlings with a nourishing solution containing 2 mM Cd, Cd +Sm: Watering the wheat seedlings with a nourishing solution containing 2 mM Cd + foliar spray with 0.5 mM silymarin, Cd+CFE: Watering the wheat seedlings with a nourishing solution containing 2 mM Cd + foliar spray with 2% clove fruit extract, Cd+CFE-Sm: Watering the wheat seedlings with a nourishing solution containing 2 mM Cd + foliar spray with clove fruit extract enriched with silymarin (0.24 g Sm L^-1^ of CFE).

### 3.2 The response of free proline, soluble sugars, and oxidative stress markers to CFE and/or silymarin

**Figure 2:**
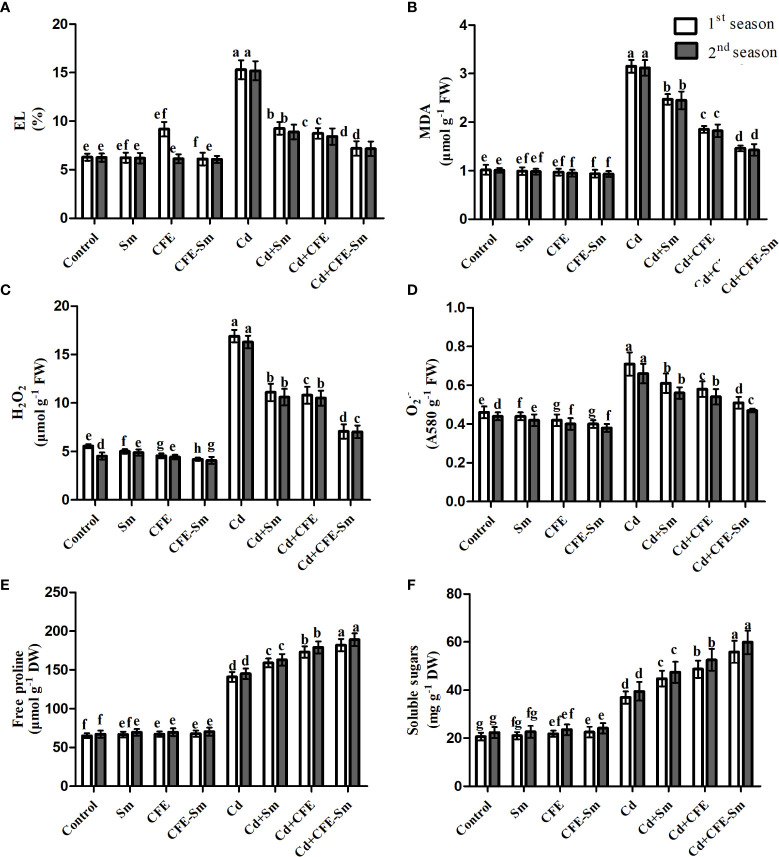
Impacts of silymarin (Sm), clove fruit extract (CFE), or silymarin-enriched clove fruit extract (CFE-Sm) foliar application on **(A)** Electrolyte leakage (EL), **(B)** malondialdehyde (MDA), **(C)** hydrogen peroxide (H_2_O_2_), **(D)** superoxide radical ( ${\text{O}}_{2}^{•-}$ ), **(E)** free proline and **(F)** soluble sugars.

Free proline and soluble sugars were increased while levels of EL, MDA, H_2_O_2,_ and ${\text{O}}_{2}^{•-}$ were slightly decreased with 2% CFE, 0.5 mM silymarin, or even with CFE-silymarin, which was the best treatment, compared to the standard control (**Table S2**; **Figure 2**). The free proline, soluble sugars, EL, MDA, H_2_O_2_ and ${\text{O}}_{2}^{•-}$ were significantly (p ≤ 0.05) stimulated under the treatment of 0.5 mM Cd compared to the standard control. These increases were 116, 78.1, 143, 209, 232 and 52.1%, respectively (**Table S2**; **Figure 2**). Compared to the stressed control treatment, EL, MDA, H_2_O_2,_ and ${\text{O}}_{2}^{•-}$ were significantly (p ≤ 0.05) inhibited under the treatment of CFE or silymarin; however, CFE-silymarin was the best treatment, which decreased all of these parameters by 52.9, 53.9, 57.5 and 28.4%, respectively. However, proline and soluble sugars increased by 29.6 and 51.5%, respectively. In addition, El and MDA levels were significantly (p ≤ 0.05) reduced in response to CFE-silymarin addition as a foliar spray compared to the stressed control treatment (**Table S2**; **Figure 2**).

### 3.3 The response of enzymes activities peroxidase, catalase, α-tocopherol, glutathione, nonenzymatic antioxidant compounds ascorbate, and superoxide dismutase

**Figure 3:**
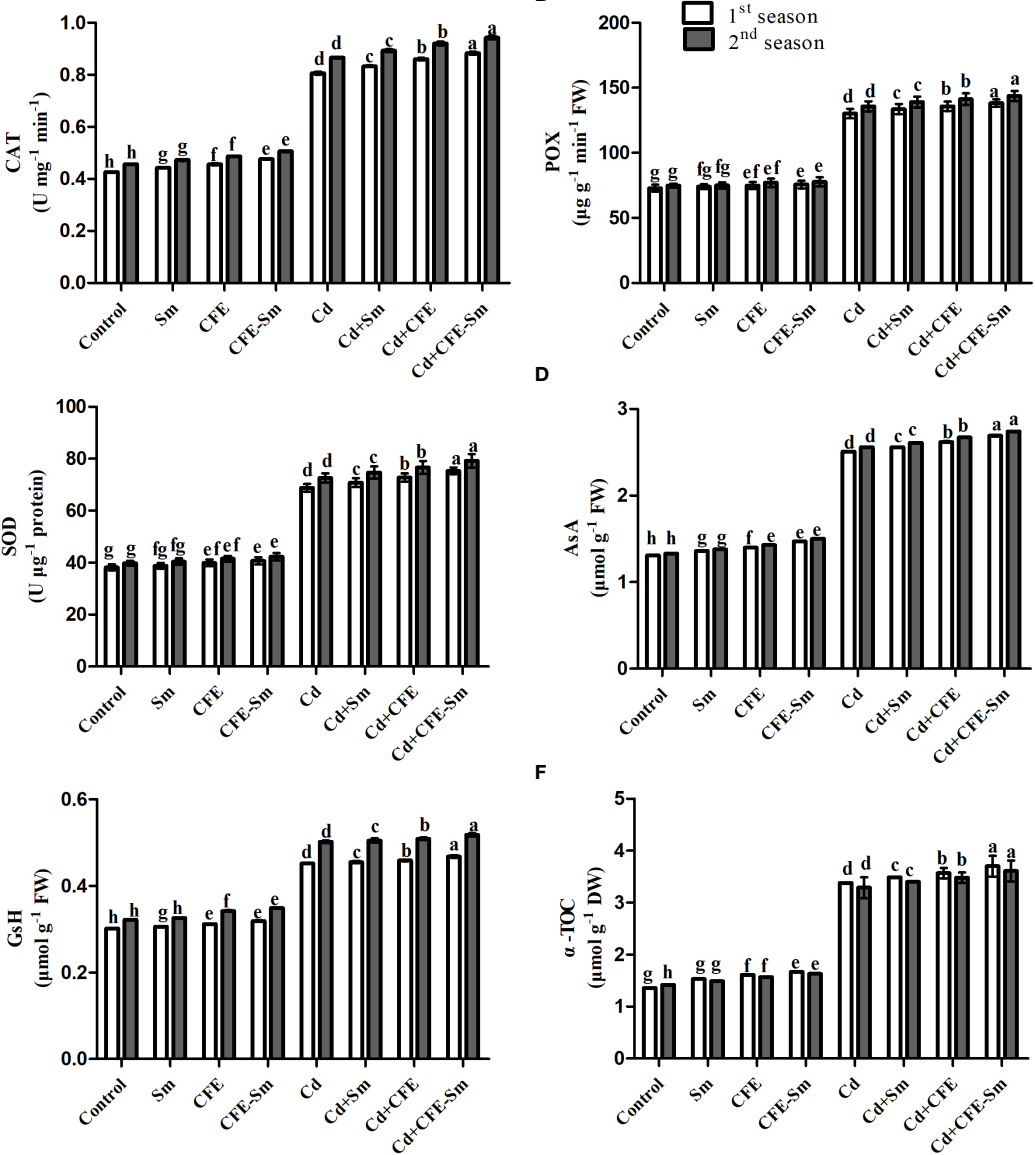
Impacts of silymarin (Sm), clove fruit extract (CFE), or silymarin-enriched clove fruit extract (CFE-Sm) foliar application on antioxidant enzymes **(A)** catalase; CAT, peroxidase; **(B)** POX, superoxide dismutase; **(C)** SOD and ascorbate peroxidase), **(D)** ascorbate (AsA), **(E)** glutathione (GSH), and **(F)** Tocopherol (-TOC) in wheat under Cd stress.

In the non-stress conditions, POX, CAT, SOD, AsA, α-TOC, and GSH were slightly elevated with 2% CFE, 0.5 mM silymarin, and also with CFE-silymarin, which had more efficiency, compared to the control (**Table S3**; **Figure 3**). Under Cd stress conditions, POX, CAT, SOD, AsA, α-TOC, and GSH were markedly elevated in average by 89.5, 79.8, 81.2, 92, 52.7 and 140% respectively when compared to the control. POX, CAT, SOD, AsA, α-TOC, and GSH were significantly (p ≤ 0.05) improved with CFE or silymarin compared to control under Cd stress. However, CEF-silymarin had more efficiency, with all of the above parameters increasing on average by 9.22, 6.01, 9.26, 7.10, 3.35, and 9.59%, respectively. Wheat plants exposed to Cd stress could increase the activities of their enzymes and improve the activity of several antioxidants to cope with markers of oxidative stress when treated with CFE-silymarin as a foliar fertilizer (**Table S3**, **Figure 3**).

### 3.4 The response of nutrient content

The contents of N, P, K, and Ca significantly (p ≤ 0.05) enhanced under the treatment of 2% CFE or 0.5 mM silymarin compared to the standard control, while Cd was not detected under normal conditions (**Table 3**). The concentration of N, P, K, and Ca (p ≤ 0.05) decreased by 24.1, 39.9, 24.1, and 50.7 under Cd stress compared to control, while Cd concentration increased. On the other hand, applying % CFE, 0.5 mM silymarin, or even CFE-silymarin alleviated the nutrient content under the Cd stress condition, while CFE-silymarin gave the highest value.

**Table 3:** N, Nitrogen; P, phosphorus; P, potassium; Ca, calcium; and Cd, cadmium; response to foliar application of Sm, silymarin; CFE, clove fruit extract; or CFE-Sm, silymarin-enriched clove fruit extract; under Cd stress.

Treatment	N (%)	P (%)	K (%)	Ca (%)	Cd (mg kg^−1^ DW)	
1^st^ season						
Control	2.20 ± 0.12^b^	0.496 ± 0.02^c^	1.86 ± 0.09^c^	2.05 ± 0.13^c^	Nd	
Sm	2.36 ± 0.16^a^	0.580 ± 0.03^b^	2.00 ± 0.11^b^	2.15 ± 0.15^b^	Nd	
CFE	2.39 ± 0.18^a^	0.593 ± 0.03^b^	2.02 ± 0.12^b^	2.20 ± 0.21^b^	Nd	
CFE-Sm	2.42 ± 0.11^a^	0.613 ± 0.04^a^	2.14 ± 0.14^a^	2.29 ± 0.16^a^	Nd	
Cd	1.68 ± 0.09^e^	0.300 ± 0.01^g^	1.37 ± 0.08^f^	1.01 ± 0.09^g^	43.9 ± 2.3^a^	
Cd+Sm	1.99 ± 0.07^d^	0.420 ± 0.02^f^	1.62 ± 0.06^e^	1.31 ± 0.06^f^	12.6 ± 1.3^b^	
Cd+CFE	2.10 ± 0.11^c^	0.450 ± 0.03^e^	1.64 ± 0.04^e^	1.43 ± 0.04^e^	7.33 ± 1.5^c^	
Cd+CFE-Sm	2.16 ± 0.10^b^	0.476 ± 0.03^d^	1.70 ± 0.05^d^	1.58 ± 0.05^d^	3.69 ± 0.21^d^	
**ANOVAdf** **Foliar 7 **	<0.001	<0.001	<0.001	<0.001	<0.001	<0.001
2^nd^ season						
Control	2.27 ± 0.13^b^	0.520 ± 0.04^c^	1.81 ± 0.06^c^	2.11 ± 0.15^c^	Nd	
Sm	2.41 ± 0.15^a^	0.603 ± 0.05^b^	2.08 ± 0.08^b^	2.21 ± 0.12^b^	Nd	
CFE	2.43 ± 0.13^a^	0.606 ± 0.06^ab^	2.10 ± 0.09^b^	2.26 ± 0.16^b^	Nd	
CFE-Sm	2.45 ± 0.16^a^	0.633 ± 0.04^a^	2.19 ± 0.13^a^	2.35 ± 0.13^a^	Nd	
Cd	1.71 ± 0.08^c^	0.310 ± 0.02^f^	1.41 ± 0.05^f^	1.04 ± 0.03^g^	43.1 ± 3.2^a^	
Cd+Sm	2.03 ± 0.09^c^	0.443 ± 0.03^e^	1.66 ± 0.03^e^	1.33 ± 0.02^f^	12.0 ± 1.5^b^	
Cd+CFE	2.20 ± 0.13^b^	0.473 ± 0.03^d^	1.68 ± 0.04^de^	1.46 ± 0.04^e^	6.84 ± 0.23^c^	
Cd+CFE-Sm	2.21 ± 0.15^b^	0.490 ± .0.02^d^	1.73 ± 0.05^d^	1.62 ± 0.05^d^	3.26 ± 0.11^d^	
**ANOVAdf** **Foliar 7 **	<0.001	<0.001	<0.001	<0.001	<0.001	<0.001

Data are means (n = 9) ± SE. The same letters in each column indicate no significant differences according to the LSD test (p ≤ 0.05).Control: There is no stress and no foliar applications, Sm: Foliar spray with 0.5 mM silymarin, CFE: Foliar spray with 2% clove fruit extract, CFE-Sm: Foliar spray with clove fruit extract enriched with silymarin (0.24 g Sm L^-1^ of CFE), Cd^+^: Watering the wheat seedlings with a nourishing solution containing 2 mM Cd, Cd +Sm: Watering the wheat seedlings with a nourishing solution containing 2 mM Cd + foliar spray with 0.5 mM silymarin, Cd+CFE: Watering the wheat seedlings with a nourishing solution containing 2 mM Cd + foliar spray with 2% clove fruit extract, Cd+CFE-Sm: Watering the wheat seedlings with a nourishing solution containing 2 mM Cd + foliar spray with clove fruit extract enriched with silymarin (0.24 g Sm L^-1^ of CFE).

### 3.5 Response of leaf feature to Cd stress and CFE and/or silymarin

**Figure 4:**
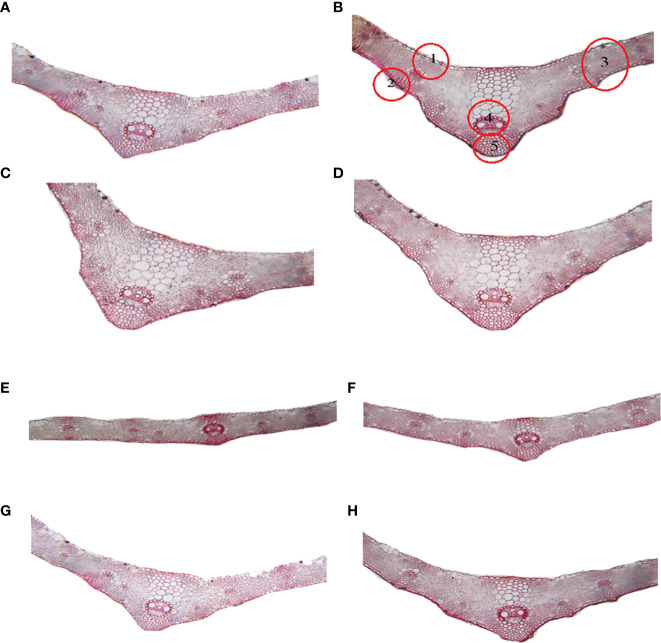
Transverse sections in the flag leaf blade on the main wheat stem as affected by foliar application of silymarin (Sm), clove fruit extract (CFE), or silymarin-enriched clove fruit extract (CFE-Sm) under Cd stress. **(A)** control, **(B)** treated with Sm, **(C)** treated with CFE, **(D)** treated with CFE-Sm, **(E)** treated with Cd, **(F)** treated with Sm + Cd, **(G)** treated with CFE + Cd, **(H)** treated with CFE-Sm + C1: Upper Epidermis, 2: Lower Epidermis, 3: Mesophyll tissue, 4: Vascular bundle of midvein, 5: Schalaranchyma tissue.

Leaf features such as blade thickness, mesophyll tissue thickness, midvein length, midvein width, vascular bund midvein width, vascular bund midvein, and thickness and width of schalaranchyma tissue were slightly increased in non-stress conditions with the treatment of 2% CFE, 0.5 mM silymarin, and CFE-silymarin, which was more efficient than the control (**Table 4**; **Figure 4**). Under Cd stress conditions; leaf features were markedly decreased compared to the control. The leaf features of the plants treated with CFE or silymarin were improved significantly compared with the control under Cd stress. However, CEF-silymarin had more efficiency, with all of the above-studied parameters, which increased by 37.3, 55.5, 100, 140, 64.2, 50, 9.47, and 11.38%, respectively (**Table 4**; **Figure 4**).

**Table 4:** Measurement in microns of certain light microscopically features of a transverse section through the leaf blade from the apex of a wheat plant (fully expanded leaf) as affected by foliar application of silymarin (Sm), clove fruit extract (CFE), or silymarin-enriched clove fruit extract (CFE-Sm) under Cd stress during the second season.

Treatment	Thickness of Blade	The thickness of mesophyll tissue	Length of midvein	Width of midvein	Length of vascular bund midvein	Width of vascular bund midvein	The thickness of schalaranchyma tissue	Width of schalaranchyma tissue
Control	202.1 ± 3.8^d^	159.6 ± 1.8^c^	476.8 ± 4.7^d^	695.8 ± 5.9^d^	120.9 ± 1.1^d^	150.9 ± 3.5^d^	121.5 ± 2.5^d^	161.8 ± 4.5^d^
Sm	205.2 ± 3.2^c^	180.8 ± 2.4^b^	574.5 ± 4.5^c^	787.3 ± 6.9^c^	123.7 ± 2.3^c^	154.7 ± 3.8^c^	122.3 ± 2.2^c^	171.3 ± 4.8^c^
CFE	223.4 ± 4.5^b^	180.8 ± 2.7^b^	627.7 ± 5.8^b^	840.5 ± 7.2^b^	130.9 ± 1.6^b^	159.93.4 ± ^b^	135.2 ± 3.1^b^	199.8 ± 4.7^b^
CFE-Sm	226.9 ± 3.7^a^	191.5 ± 2.9^a^	670.3 ± 6.2^a^	904.4 ± 8.1^a^	133.2 ± 1.8^a^	164.2 ± 3.7^a^	146.1 ± 3.4^a^	204.8 ± 3.6^a^
Cd	134.3 ± 2.6^h^	95.7 ± 1.8^g^	234.0 ± 2.6^h^	234.1 ± 3.5^h^	66.6 ± 1.5^h^	92.8 ± 2.5^h^	101.3 ± 3.8^f^	158.1 ± 3.8^g^
Cd+Sm	156.5 ± 2.4^g^	112.4 ± 2.1^f^	257.4 ± 3.4^g^	385.6 ± 3.8^g^	87.5 ± 1.4^g^	110.6 ± 2.9^g^	103.2 ± 3.8^g^	158.9 ± 3.4^g^
Cd+CFE	180.8 ± 2.7^f^	138.3 ± 1.8^e^	436.2 ± 4.1^f^	559.6 ± 4.5^f^	106.6 ± 2.4^f^	137.5 ± 3.1^f^	107.5 ± 1.6^f^	168 ± 2.6^f^
Cd+CFE-Sm	184.4 ± 2.9^e^	148.9 ± 2.4^d^	468.1 ± 4.8^e^	563.9 ± 5.6^e^	109.4 ± 2.9^e^	139.2 ± 3.7^e^	110.9 ± 5.2^e^	176.1 ± 5.1^e^
**ANOVAdf** **Foliar**	<0.001	<0.001	<0.001	<0.001	<0.001	<0.001	<0.001	<0.001

Data are means (n = 9) ± SE. The same letters in each column indicate not significant differences according to theLSD test (p ≤ 0.05).Control: There is no stress and no foliar applications, Sm: Foliar spray with 0.5 mM silymarin, CFE: Foliar spray with 2% clove fruit extract,CFE-Sm: Foliar spray with clove fruit extract enriched with silymarin (0.24 g Sm L^-1^ of CFE), Cd^+^: Watering the wheat seedlings with a nourishing solution containing 2 mM Cd,Cd +Sm: Watering the wheat seedlings with a nourishing solution containing 2 mM Cd + foliar spray with 0.5 mM silymarin,Cd+CFE: Watering the wheat seedlings with a nourishing solution containing 2 mM Cd + foliar spray with 2% clove fruit extract, Cd+CFE-Sm: Watering the wheat seedlings with a nourishing solution containing 2 mM Cd + foliar spray with clove fruit extract enriched with silymarin (0.24 g Sm L^-1^ of CFE).

## 4 Discussion

Cd is considered one of the most highly toxic and mobile heavy metals in soil and plant, owing to its obstruction of many physio-biochemical and molecular processes (Desoky et al., 2020a; Jung et al., 2021). In the present study, adding a Cd-containing nutrient solution to wheat plants hampered growth and productivity (**Table 2**) and reduced photosynthetic pigments and photosynthetic efficiency (**Figure 1**), along with increased oxidative stress inducers (H_2_O_2_ and ${\text{O}}_{2}^{•-}$ ), lipid peroxidation (MDA) and EL (**Figure 2**). Cd degrades chlorophyll, restricts nutrient uptake, and reduces photosynthetic activity due to Cd toxicity-induced oxidative burst and over-ROS accumulation in plant cells, leading to reducing plant performance (Alzahrani et al., 2018; Haider et al., 2022). Furthermore, Cd toxicity may decrease dry matter accumulation due to the inhibition of root growth caused by Cd accumulation at a higher rate in roots than in shoots (Ali et al., 2022; Zeshan et al., 2022).

Nonetheless, our research demonstrated that applying CFE and silymarin mediated positive changes to relieve Cd toxicity in wheat plants. The highest positive effects were assigned to co-apply CFE-silymarin, yielding the best results. Exogenous application of CFE and silymarin improved the growth and yield of Cd-stressed wheat plants (**Table 2**). These findings may be due to the fact that CFE and silymarin applications associated with decreased Cd contents (**Figure 3**), and H_2_O_2_ and ${\text{O}}_{2}^{•-}$ (**Figure 2**), which are proposed to participate in maintaining membrane integrity, cell turgor status, reduced lipid peroxidation, and EL from the cells under Cd toxicity (**Figures 1**, **2**) (Rady and Mohamed, 2015). CFE analysis ensured the existence of the flavonoids and phenolic components (i.e., Ellagic-acid, 3,4 Dihydroxybenzoic-acid, Eugenyl-acetate, Eugenol, Gallic-acid, tannic acid, and Naphthalene), osmoprotectants (i.e., proline, glutathione, and soluble sugars), nutrients (i.e., N, K, P, Ca, Mg, Fe), vitamins (i.e., A, E, D, C) and antioxidants (**Table 1**). As a result of its diverse composition, CFE was proposed as a plant biostimulator. The enhanced mobilization of inorganic metabolites solutes like ascorbic acid (Vitamin C), K, and Ca present in CFE (**Table 1**) to the growing plumule and/or the increase in amylase activity and reducing sugars, contributing to early vigor and increased plant growth, may explain the improved plant growth traits (i.e., area of leaves per plant, dry weight, and shoot fresh) caused by CFE application (Afzal et al., 2012; Desoky et al., 2021b). Also, the phenolic component is critical to decline the damaging effects of drought stress in the plant (Naikoo et al., 2019). The different physiological processes correlated to development and growth in plants, including the synthesis of photosynthetic pigments, cell division, and seed germination, were influenced by phenolic compounds (Tanase et al., 2019). Phenolics have been utilized for various applications, such as antioxidants as food additives, allelochemical bioremediation, and plant growth promotion (Bujor et al., 2015). The accumulation of phenolic compounds is generally a consistent feature of plants subjected to stress, which is considered a defensive mechanism to face various abiotic stresses (Cheynier et al., 2013). Phenolic compounds influence many physiological processes in plants, increasing the adaptability and tolerance of plants subjected to suboptimal conditions (Andersen, 2003). Flavonols’ accumulation and biosynthesis were also catalyzed in plants subjected to water deficit conditions, accompanied by improved drought stress resistance (Ballizany et al., 2012). The accumulation of flavonoids in cytoplasm plays a critical role in detoxifying and reducing the harmful effect of H_2_O_2_ molecules generated by the stress, and in the end, oxidation of flavonoids is followed by ascorbic acid-mediated re-conversion of flavonoids into primary metabolites (Hernandez et al., 2009). However, under stress, exogenous Gallic-acid (GLA) treatment resulted in the elevation of plant growth (Ozfidan-Konakci et al., 2015).In addition, the increased CFE contains macro elements that alleviate stress’s reverse effect on growth (Yildirim et al., 2009). Erner et al. (2002) indicated that applying K mitigated the negative impact of stress on plant growth. Also, Martinez and Cerda (1999) reported that foliar spray with N alone ameliorated the stress effect on plant growth. On the other hand, CFE contains microelements that can enhance plant tolerance to drought by improving root growth and promoting nutrient uptake (Abou El-Nour, 2002). In addition,silymarin-fortified biostimulants improved crop growth and productivity by regulating adaptive mechanisms *via* modulation of physio-biochemical and molecular processes under Cd stress (Ali et al., 2022).

Cd uptake and accumulation in plants produce ROS by substituting cofactors for basic metal ions in metalloproteins *via* the Fenton reaction, which destroys membrane cells *via* lipid peroxidation and prevents the biosynthesis of cellular molecules necessary for plant growth (Jung et al., 2021). In this study, an increase in H_2_O_2_ and ${\text{O}}_{2}^{•-}$ of Cd-stressed wheat plants was observed in tandem with an increase in EL and lipid peroxidation of membranes (determined as MDA). These unfavorable results were improved by foliage-applied CFE and silymarin, which reduced the concentrations of these free radicals, lipid peroxidation, and EL, indicating that co-applied CFE and silymarin improved Cd tolerance. CFE and silymarin co-application was a powerful catalyst for growing wheat against Cd stress, as it enhanced photosynthetic capacity (i.e., chlorophyll content and photosynthetic efficiency), providing wheat plants with more photo-assimilates needed to produce dry biomass and grain yield (Bel et al., 2003). These improvements in the photosynthetic efficacy due to the role of CFE and silymarin reduced inner Cd content in different wheat parts (roots, shoots, and grain). They elevated the antioxidative defense system for suppressing the oxidative stress markers. Bioactive compounds in silymarin, such as flavonoids and polyphenols, have the antioxidant capacity for scavenging ROS, decreasing free radicals levels, and protecting plant tissue from oxidative stress caused by Cd toxicity (de Oliveira et al., 2015; Ali et al., 2022).

Furthermore, the CFE application enhanced the photosynthetic efficiency of wheat plants, possibly due to increased growth for better water and nutrient uptake (Desoky et al., 2021b). The increase in the photosynthetic attributes of CFE under stress may be due to its effectiveness in reducing the MDA and H_2_O_2_ contents. Presence nutrients in CFE inhibit premature leaf senescence and maintain higher leaf area, increasing photosynthetic pigments. In addition, Fe found in CFE may be available in plants after treatment to activate many enzymes involved in the chlorophyll biosynthesis pathway and some antioxidant enzymes such as APOX and GR that scavenge the ROS and protect chlorophyll from degradation (Zayed et al., 2011).

Plant defense mechanisms, such as the accumulation of antioxidative compounds, control the level of free radicals under normal conditions. Under Cd stress, the biosynthesis and accumulation of osmoadaptive compounds such as proline, soluble sugar, and antioxidant-related substances such as phenolic, AsA, GSH,α-TOC, and enzymatic antioxidants protect stressed plants by modulating osmotic pressure and suppressing Cd-induced oxidative stress (Gill and Tuteja, 2010). In the present study, we observed an increment in the proline, soluble sugar, AsA, α-TOC, and GSH contents in Cd-stressed wheat plants in response to the integrative application of CFE and silymarin. Proline accumulates in stressed plant tissues to act as an osmoprotectant for osmotic adjustment and is directly involved in ROS detoxification, cell structure maintenance, lipid membrane oxidation-reduction, and photoinhibition (Semida et al., 2020). This increased proline accumulation may be related to CFE and silymarin-induced significant modulation in proline metabolism *via* decreased P5CS anabolism and increased ProDH catabolism in order to balance the concentration of proline within plant cells (Alharby et al., 2020). The results presented here show an increase in AsA,α-TOC, and GSH levels in Cd-stressed wheat plants treated with CFE and silymarin, indicating an improvement in the AsA-GSH cycle with a decisive role in detoxifying ROS and wheat tolerance (Hasanuzzaman et al., 2019). Furthermore, increased GSH levels promote phytochelatin formation and Cd ion sequestration in the vacuole *via* complex phytochelatin formation (Elrys et al., 2021).

Kapoor et al. (2015) state that the antioxidative defense machinery usually regulates cellular redox homeostasis by controlling the ROS level in the cells. Therefore, and as evidenced herein, it is assumed that the activity of antioxidant enzymes will increase in Cd-stressed plants. Moreover, externally applied CFE and/or silymarin to Cd-stressed wheat plants further enhanced antioxidant enzymes (**Table S3**; **Figure 3**). CAT, POX, and SOD activity in this research was up-regulated against the free radicals induced by Cd toxicity. This elevated enzyme activity reached its maximum by co-applicating CFE and silymarin. Our findings are consistent with those of Alharby et al. (2021); Ali et al. (2022), and Desoky et al. (2021b), who reported that CFE and/or silymarin improved cell metabolism under Cd stress by up-regulating the activity of antioxidant enzymes such as SOD, CAT, and POX synchronized with reduced Cd content, boosting the plant’s capacity to resist Cd stress.

Finally, the role of silymarin or CFE as a secondary metabolite in enhancing plant growth and production under Cd stress conditions was still unclear. Our findings evidenced that silymarin can increase the wheat quantity and quality under Cd stress conditions by improving wheat plant defenses. Our study is in line with the previous reports that considered silymarin as a powerful antioxidant, increasing plant resistance to stress (Ghavami and Ramin, 2008; Afshar et al., 2015). The CFE-silymarin results in our study align with the properties of the biostimulator reported by the European Biostimulant Industry Council (Colla and Rouphael, 2015) and with the findings of other work examining different stresses (Alharby et al., 2020). The bioactive compounds included in CFE-silymarin make it an effective biocatalyst and a unique environmentally friendly strategy. CFE-silymarin bioactive ingredients have different antioxidants, which possess high states of redox, making CFE-silymarin possess the ability to suppress ROS and lipid peroxidation. Accordingly, our study suggested that the interplaying of the bioactive ingredients of CFE-silymarin formed a robust defense system against Cd-induced oxidative damage in favor of wheat plants’ performance.

## 5 Conclusions

Plant height, shoot dry weight, leaf area, No grain spike, 1000 grain weight, grain yield, total chlorophylls, total carotenoids, Pn, Tr, Gs, RWC, and MSI were all reduced by 41.8, 60.6, 56.7, 55.9, 50.4, 82.8, 33.5, 23.8, 41.2, 48.7, 48.1, 22.7, and 42.5%, respectively, under the stressed control (0.5 mM Cd) treatment. Compared to the stressed control, the above attributes were significantly enhanced with the treatment of CFE and/or silymarin. In contrast, CFE-silymarin treatment was the most efficient treatment, as all of the above attributes enhanced by 60.4, 97.1, 86.7, 94.1, 87.2, 160.9, 44.2, 22.2, 56.2, 57.1, 69.7, 22.2, and 63.3%, respectively. In contrast, the levels of free proline, soluble sugars, EL, MDA, H_2_O_2_, ${\text{O}}_{2}^{•-}$ POX, CAT, SOD, AsA, α-TOC, and GSHwere significantly stimulated by 116, 78.1, 143, 209, 232, 52.1, 89.5, 79.8, 81.2, 92, 52.7 and 140%, respectively under the treatment of 0.5 mM Cd compared to the standard control. Compared to the stressed control treatment, EL, MDA, H_2_O_2,_ and ${\text{O}}_{2}^{•-}$ were significantly inhibited under the treatment of CFE or silymarin; however, CFE-silymarin was the best treatment, which decreased these parameters by 52.9, 53.9, 57.5 and 28.4%, respectively, while increased POX, CAT, SOD, AsA, α-TOC, and GSH by 9.22, 6.01, 9.26, 7.10, 3.35, and 9.59%, respectively. The findings of our study indicate that CFE-silymarin spraying on wheat plants under Cd stress is an efficient method to enhance the buildup of biomass and plant development. In our investigation, the antioxidant components of CFE-silymarin served as a natural biostimulant that interacted with one another to benefit wheat plants exposed to Cd stress. Foliar spraying wheat plants with CFE-silymarin improved growth, photosynthetic efficiency, nonenzymatic and enzymatic antioxidants. This also reduced oxidative damage caused by ROS ( ${\text{O}}_{2}^{•-}$ and H_2_O_2_), ionic leakage, and lipid peroxidation. The limitation of Cd ion buildup and the activation of antioxidant defenses in wheat plants under Cd stress led to these adequate findings. The results of this work suggest using CFE-silymarin as a potent novel biostimulator for wheat to stimulate various physiological and metabolic processes and increase wheat plants’ tolerance to Cd stress. In order to fully understand the mechanism of silymarin (in CFE-silymarin) for stress-tolerant plants, extensive research is required.

## Data availability statement

The original contributions presented in the study are included in the article/**Supplementary Material**. Further inquiries can be directed to the corresponding authors.

## Author contributions

Conceptualization: E-SD, JL, HA, MR and AE-S. Methodology: E-SD. Software: E-SD, LW, and AE-S. Validation: HA and MI. Formal analysis, E-SD, LW, MM, and AE-S. Investigation: E-SD, AE-S, and MM. Resources: E-SD, MR, MM. Writing—original draft preparation: E-SD and MM. Writing—review and editing: XZ, HA, LW, MI, KY, MR, PM, E-SD and AE-S. All authors contributed to the article and approved the submitted version.
