# Supplementary material for: Interplay of silymarin and clove fruit extract effectively enhances cadmium stress tolerance in wheat (Triticum aestivum)
Source: Front Plant Sci. 2023 Apr 14;14:1144319. doi: 10.3389/fpls.2023.1144319 (PMC10140571; doi:10.3389/fpls.2023.1144319)
Supplement: Supplementary file 1 [file Table_1.docx]

**Table** S**1**. Physiological parameters of wheat plant under Cd stress.
Leaf photosynthetic pigments (total chlorophylls and total carotenoids), photosynthetic efficiency (net photosynthetic rate, Pn; transpiration rate, Tr; and Stomatal conductance, Gs), relative water content (RWC), and membrane stability index (MSI) after foliar application of silymarin (Sm), clove fruit extract (CFE), or silymarin-enriched clove fruit extract (CFE-Sm).

| Treatment | Total chlorophylls (mg g^‒1^FW) | Total carotenoids (mg g^‒1^FW) | Net photosynthetic rate ( µmol CO_2_ m^‒2^ s^‒1^) | Transpiration rate ( mMol H_2_O m^‒2^ s^‒1^) | Stomatal conductance (mMol H_2_O m^‒2^ s^‒1^) | RWC  (%) | MSI  (%) |
| --- | --- | --- | --- | --- | --- | --- | --- |
| **1^st^ season** | | | | | | | |
| Control | 2.12±.07^c^ | 0.813±.07^c^ | 10.0±0.6^c^ | 6.0±.08^d^ | 0.416±.03^d^ | 70.3±2.6^d^ | 53.5±2.2^d^ |
| Sm | 2.15±.08^bc^ | 0.816±.08^b^ | 10.9±0.7^b^ | 6.20±.09^c^ | 0.440±.03^c^ | 72.4±2.9^c^ | 58.1±2.6^c^ |
| CFE | 2.19±.05^ab^ | 0.836±.09^a^ | 11.2±0.9^b^ | 6.30±.07^b^ | 0.460±.02^b^ | 73.6±3.1^b^ | 59.4±2.7^b^ |
| CFE-Sm | 2.23±.04^a^ | 0.846±.08^a^ | 14.4±1.1^a^ | 6.40±.05^a^ | 0.486±.03^a^ | 74.7±3.4^a^ | 61.2±2.4^a^ |
| Cd | 1.42±.01^g^ | 0.620±.04^f^ | 5.91±0.3^g^ | 3.20±.02^h^ | 0.223±.01^h^ | 54.7±1.4^h^ | 31.6±1.1^h^ |
| Cd+Sm | 1.75±.03^f^ | 0.730±.05^e^ | 8.32±0.3^f^ | 4.60±.02^g^ | 0.326±.02^g^ | 62.6±1.9^g^ | 44.9±1.4^g^ |
| Cd+CFE | 1.91±.04^e^ | 0.743±.06^d^ | 8.74±0.7^e^ | 4.70±.03^f^ | 0.346±.02^f^ | 64.3±1.8^f^ | 47.6±1.6^f^ |
| Cd+CFE-Sm | 2.04±.03^d^ | 0.760±.06^c^ | 9.29±0.7^d^ | 4.91±.03^e^ | 0.366±.03^e^ | 66.3±1.7^e^ | 51.4±1.9^e^ |
| **2^nd^ season** | | | | | | | |
| Control | 2.20±.06^c^ | 0.843±.08^b^ | 10.8±0.6^c^ | 6.61±0.2^d^ | 0.496±.03^d^ | 71.6±3.6^d^ | 57.2±2.6^d^ |
| Sm | 2.23±.07^bc^ | 0.846±.09^b^ | 11.7±0.8^b^ | 6.80±0.3^c^ | 0.520±.04^c^ | 73.7±3.8^c^ | 59.0±2.9^c^ |
| CFE | 2.27±.06^ab^ | 0.866±.08^a^ | 12.0±1.2^b^ | 6.90±0.4^b^ | 0.540±.04^b^ | 75.0±3.9^b^ | 60.4±2.7^b^ |
| CFE-Sm | 2.32±.07^a^ | 0.876±.07^a^ | 14.8±1.4^a^ | 7.01±0.5^a^ | 0.566±.03^a^ | 76.1±3.5^a^ | 62.2±2.8^a^ |
| Cd | 1.45±.02^g^ | 0.640±.04^f^ | 6.31±0.5^g^ | 3.24±.06^h^ | 0.243±.01^h^ | 54.8±2.1^h^ | 31.9±1.5^h^ |
| Cd+Sm | 1.91±.02^f^ | 0.750±.04^e^ | 8.92±0.8^f^ | 4.92±.07^g^ | 0.386±.02^g^ | 63.8±2.4^g^ | 45.8±2.5^g^ |
| Cd+CFE | 1.96±.03^e^ | 0.763±.03^d^ | 9.34±0.9^e^ | 5.02±0.2^f^ | 0.406±.01^f^ | 65.5±2.7^f^ | 46.6±2.6^f^ |
| Cd+CFE-Sm | 2.10±.01^d^ | 0.780±.04^c^ | 9.8±0.8^d^ | 5.21±0.3^e^ | 0.426±.02^e^ | 67.6±1.9^e^ | 52.3±2.7^e^ |

Data are means (n = 9) ± SE. The same letters in each column indicate not significant differences according to the LSD test (*p* ≤ 0.05). **Control**: There is no stress and no foliar applications, **Sm**: Foliar spray with 0.5 mM silymarin, **CFE**: Foliar spray with 2% clove fruit extract, **CFE-Sm**: Foliar spray with clove fruit extract enriched with silymarin (0.24 g Sm L^-1^ of CFE), **Cd**^+^: Watering the wheat seedlings with a nourishing solution containing 2 mM Cd^2+^, **Cd +Sm**: Watering the wheat seedlings with a nourishing solution containing 2 mM Cd^2+^ + foliar spray with 0.5 mM silymarin, **Cd +CFE**: Watering the wheat seedlings with a nourishing solution containing 2 mM Cd^2+^ + foliar spray with 2% clove fruit extract, **Cd +CFE-Sm**: Watering the wheat seedlings with a nourishing solution containing 2 mM Cd^2+^ + foliar spray with clove fruit extract enriched with silymarin (0.24 g Sm L^-1^ of CFE).
