# Supplementary material for: Interplay of silymarin and clove fruit extract effectively enhances cadmium stress tolerance in wheat (Triticum aestivum)
Source: Front Plant Sci. 2023 Apr 14;14:1144319. doi: 10.3389/fpls.2023.1144319 (PMC10140571; doi:10.3389/fpls.2023.1144319)
Supplement: Supplementary file 2 [file Table_2.docx]

**Table** **2**. Electrolyte leakage (EL), malondialdehyde (MDA), hydrogen peroxide (H_2_O_2_) and superoxide radical (O_2_ ^•-^ ), free proline and soluble sugars to foliar application of silymarin (Sm), clove fruit extract (CFE), or silymarin-enriched clove fruit extract (CFE-Sm) of wheat plants under Cd stress.

| Treatment | EL (%) | MDA  (μ mol/g FW) | H_2_O_2_ (µmol g^‒1^ FW) | O_2_^•−^ (A580 g^−1^ FW) | Free proline (µmol g^‒1^ DW) | Soluble sugars (mg g^‒1^ DW) |
| --- | --- | --- | --- | --- | --- | --- |
| **1^st^ season** | | | | | | |
| Control | 6.29±0.36^e^ | 1.02±0.10^e^ | 5.55±0.21^e^ | 0.46±0.03^e^ | 65.2±3.3^f^ | 20.6±1.6^g^ |
| Sm | 6.24±0.52^ef^ | 0.99±0.08^ef^ | 5.01±0.23^f^ | 0.44±0.02^f^ | 66.7±3.7^ef^ | 21.0±1.5^fg^ |
| CFE | 9.17±0.75^ef^ | 0.97±0.07^ef^ | 4.55±0.25^g^ | 0.42±0.03^g^ | 67.1±3.5^e^ | 21.8±1.3^ef^ |
| CFE-Sm | 6.11±0.65^f^ | 0.94±0.08^f^ | 4.20±0.16^h^ | 0.40±0.02^g^ | 67.8±4.2^e^ | 22.5±2.2^e^ |
| Cd | 15.3±0.98^a^ | 3.15±0.13^a^ | 16.9±0.65^a^ | 0.71±0.06^a^ | 141±6.3^d^ | 36.9±2.6^d^ |
| Cd+Sm | 9.26±0.65^b^ | 2.47±0.11^b^ | 11.1±0.89^b^ | 0.61±0.05^b^ | 159±5.6^c^ | 44.8±3.2^c^ |
| Cd+CFE | 8.74±0.55^c^ | 1.85±0.07^c^ | 10.8±0.88^c^ | 0.58±0.04^c^ | 173±7.3^b^ | 48.7±3.6^b^ |
| Cd+CFE-Sm | 7.19±0.75^d^ | 1.46±0.06^d^ | 7.07±0.74^d^ | 0.51±0.03^d^ | 182±7.8^a^ | 55.9±4.6^a^ |
| **2^nd^ season** | | | | | | |
| Control | 6.25±0.42^e^ | 1.01±0.05^e^ | 4.53±0.36^d^ | 0.44±0.02^d^ | 67.1±4.6^f^ | 22.3±2.3^g^ |
| Sm | 6.19±0.53^e^ | 0.98±0.06^ef^ | 4.89±0.32^e^ | 0.42±0.03^e^ | 69.4±4.4^e^ | 22.6±2.5^fg^ |
| CFE | 6.13±0.46^e^ | 0.95±0.07^f^ | 4.43±0.23^e^ | 0.40±0.03^f^ | 69.7±4.9^e^ | 23.5±2.3^ef^ |
| CFE-Sm | 6.07±0.36^e^ | 0.93±0.06^f^ | 4.08±0.36^g^ | 0.38±0.02^f^ | 70.4±5.3^e^ | 24.1±2.2^e^ |
| Cd | 15.2±0.98^a^ | 3.12±0.16^a^ | 16.3±0.65^a^ | 0.66±0.05^a^ | 145±6.8^d^ | 39.5±3.9^d^ |
| Cd+Sm | 8.89±0.75^b^ | 2.45±0.18^b^ | 10.6±0.85^b^ | 0.56±0.03^b^ | 163±7.5^c^ | 47.4±4.4^c^ |
| Cd+CFE | 8.42±0.84^c^ | 1.82±0.13^c^ | 10.5±0.78^b^ | 0.54±0.04^b^ | 179±7.7^b^ | 52.6±4.6^b^ |
| Cd+CFE-Sm | 7.15±0.75^d^ | 1.43±0.12^d^ | 7.03±0.65^c^ | 0.47±0.01^c^ | 189±8.2^a^ | 59.9±4.9^a^ |

Data are means (n = 9) ± SE. The same letters in each column indicate not significant differences according to the LSD test (*p* ≤ 0.05). **Control**: There is no stress and no foliar applications, **Sm**: Foliar spray with 0.5 mM silymarin, **CFE**: Foliar spray with 2% clove fruit extract, **CFE-Sm**: Foliar spray with clove fruit extract enriched with silymarin (0.24 g Sm L^-1^ of CFE), **Cd**^+^: Watering the wheat seedlings with a nourishing solution containing 2 mM Cd^2+^, **Cd +Sm**: Watering the wheat seedlings with a nourishing solution containing 2 mM Cd^2+^ + foliar spray with 0.5 mM silymarin, **Cd +CFE**: Watering the wheat seedlings with a nourishing solution containing 2 mM Cd^2+^ + foliar spray with 2% clove fruit extract, **Cd +CFE-Sm**: Watering the wheat seedlings with a nourishing solution containing 2 mM Cd^2+^ + foliar spray with clove fruit extract enriched with silymarin (0.24 g Sm L^-1^ of CFE).
