# Supplementary material for: Interplay of silymarin and clove fruit extract effectively enhances cadmium stress tolerance in wheat (Triticum aestivum)
Source: Front Plant Sci. 2023 Apr 14;14:1144319. doi: 10.3389/fpls.2023.1144319 (PMC10140571; doi:10.3389/fpls.2023.1144319)
Supplement: Supplementary file 3 [file Table_3.docx]

**Table** S**3**. Antioxidant enzymes (catalase; CAT, peroxidase; POX, super oxide dismutase; SOD and ascorbate peroxidase), ascorbate (AsA), glutathione (GSH), and Tocopherol (-TOC) responses to foliar application of silymarin (Sm), clove fruit extract (CFE), or silymarin-enriched clove fruit extract (CFE-Sm) in wheat under Cd stress.

| Treatment | CAT  (U mg^‒1^ min^‒1^) | POX  (µg g^‒1^ fresh weight min^‒1^) | SOD  (U µg^‒1^ protein) | AsA  (µmol g^‒1^ FW) | GsH  (µmol g^‒1^ FW) | *α*-TOC (µmol g^‒1^ DW) |
| --- | --- | --- | --- | --- | --- | --- |
| **1^st^ season** | | | | | | |
| Control | 0.426±.003^h^ | 72.9±2.6^g^ | 38.2±1.2^g^ | 1.31±.009^h^ | 0.302±.001^h^ | 1.36±.002^g^ |
| Sm | 0.443±.002^g^ | 74.0±1.9^fg^ | 38.8±1.1^fg^ | 1.36±.005^g^ | 0.306±.001^g^ | 1.53±.003^g^ |
| CFE | 0.456±.004^f^ | 74.9±2.7^ef^ | 39.9±1.3^ef^ | 1.40±.006^f^ | 0.312±.001^e^ | 1.61±.001^f^ |
| CFE-Sm | 0.476±.002^e^ | 75.7±2.9^e^ | 40.7±1.4^e^ | 1.47±.005^e^ | 0.319±.002^e^ | 1.67±.002^e^ |
| Cd | 0.806±.006^d^ | 130.2±3.7^d^ | 68.8±1.5^d^ | 2.51±.007^d^ | 0.452±.001^d^ | 3.38±.006^d^ |
| Cd+Sm | 0.833±.003^c^ | 133.6±3.9^c^ | 70.8±1.7^c^ | 2.56±.006^c^ | 0.455±.003^c^ | 3.49±.007^c^ |
| Cd+CFE | 0.860±.005^b^ | 135.8±3.6^b^ | 72.8±1.6^b^ | 2.62±.008^b^ | 0.459±.002^b^ | 3.57±0.1^b^ |
| Cd+CFE-Sm | 0.883±.006^a^ | 138.2±2.8^a^ | 75.3±1.3^a^ | 2.69±.009^a^ | 0.468±.003^a^ | 3.70±0.2^a^ |
| **2^nd^ season** | | | | | | |
| Control | 0.456±.002^h^ | 74.9±1.5^g^ | 39.8±0.9^g^ | 1.33±.002^h^ | 0.322±.001^h^ | 1.42±.003^h^ |
| Sm | 0.473±.003^g^ | 75.0±2.4^fg^ | 40.4±1.2^fg^ | 1.38±.001^g^ | 0.326±.001^h^ | 1.49±.002^g^ |
| CFE | 0.487±.002^f^ | 77.0±3.2^ef^ | 41.5±1.1^ef^ | 1.43±.005^e^ | 0.342±.002^f^ | 1.57±.005^f^ |
| CFE-Sm | 0.506±.004^e^ | 77.7±3.5^e^ | 42.3±1.4^e^ | 1.50±.003^e^ | 0.349±.002^e^ | 1.63±.003^e^ |
| Cd | 0.866±.003^d^ | 135.7±3.8^d^ | 72.6±1.8^d^ | 2.56±.005^d^ | 0.502±.003^d^ | 3.29±0.2^d^ |
| Cd+Sm | 0.893±.006^c^ | 139.1±4.2^c^ | 74.7±2.3^c^ | 2.61±.007^c^ | 0.505±.005^c^ | 3.40±.009^c^ |
| Cd+CFE | 0.920±.007^b^ | 141.3±4.4^b^ | 76.6±2.5^b^ | 2.67±.003^b^ | 0.509±.003^b^ | 3.48±0.1^b^ |
| Cd+CFE-Sm | 0.943±.007^a^ | 143.7±3.9^a^ | 79.2±2.6^a^ | 2.74±.005^a^ | 0.518±.004^a^ | 3.61±0.2^a^ |

Data are means (n = 9) ± SE. The same letters in each column indicate not significant differences according to the LSD test (*p* ≤ 0.05). **Control**: There is no stress and no foliar applications, **Sm**: Foliar spray with 0.5 mM silymarin, **CFE**: Foliar spray with 2% clove fruit extract, **CFE-Sm**: Foliar spray with clove fruit extract enriched with silymarin (0.24 g Sm L^-1^ of CFE), **Cd**^+^: Watering the wheat seedlings with a nourishing solution containing 2 mM Cd^2+^, **Cd +Sm**: Watering the wheat seedlings with a nourishing solution containing 2 mM Cd^2+^ + foliar spray with 0.5 mM silymarin, **Cd +CFE**: Watering the wheat seedlings with a nourishing solution containing 2 mM Cd^2+^ + foliar spray with 2% clove fruit extract, **Cd +CFE-Sm**: Watering the wheat seedlings with a nourishing solution containing 2 mM Cd^2+^ + foliar spray with clove fruit extract enriched with silymarin (0.24 g Sm L^-1^ of CFE).
